# Prescriptions

**Published:** 1896-04

**Authors:** 


[BILIOUSNESS.

℞. Sodii sulphat.,
Potassii et sodii tart. . aa ℥ j.
Infusi cascarillæ, . . . . . . . . ℥ viij.
M. Sig :—Two tablespoonfuls three times daily.—FORTHERGILL.

BURNS AND SCALDS.

℞. Ol. lini,
Liq. calcis . . . . . . . . . . . . . . aa ℥ iv
Acidi carbolici . . . . ad gtt. xxx.
M. Sig :—Apply freely. (*Charity Hospital, N. Y.*)

LUMBAGO.

℞. Tinct. iodinii . . . . . . . . . . . . ʒ ij.

Tinct. aconiti rad . . . . . . . . ʒ iij.

Chloroformi . . . . . . . . . . . . . ʒ iv.

Linimenti saponis co. . . ad ℥ iij.

M. Sig :—Apply every few hours locally.—*Bellevue Hosp., N. Y.*

INSOMNIA.

℞. Antipyrin . . . . . . . . . . . . . . ʒ i—ij.
Syr. aurantii cort. . . . . . . ℥ j.
Aquæ cinnamomi . . . . ad ℥ iij.
M. Sig.—One tablespoonful every hour or two till effective.—WILLIAMS, *Annual Univ. Med. Sci.*

INCONTINENCE OF URINE.

℞. Tinct. ferri chlor . . . . . . . ʒ ij.

Ext. ergotæ fld . . . . . . . . . . ʒ v.

Spts. chloroformi . . . . . . . ʒ ij.

Tinct. quassiæ . . . . . . . . ad ℥ iv.

M. Sig :—A teaspoonful in a wine-glassful of water thrice daily. (*For children.*)—S. O. P[illegible].

### HYSTERIA.

℞. Ext. conii fld.,
Ext. hyoscyami fld....aa ♏ vij.
Chloral hydratis......... gr. x.
Aquæ,.................. ℥ j.
Ft. haustus.
M. Sig:—To be taken at a single dose and repeated as required. —MADIGAN, *Annual Univ. Med. Sci.*

### ALOPECIA.

℞. Quiniæ sulphat.......... ℈ iv.

Spiriti vini rectif......... ℥ iv.

Tinct. capsici,

Tinct, cantharidis,

Spts. ammon. arom....aa ℥ ss.

Glycerinæ,................ ℥ iv.

Aquæ..........q. s. ad ft. Oj.

M. Sig:--Apply locally.—BRINTON

### ANGINA PECTORIS.

℞. Antipyrin............... ʒ j.

Syr. tolutan............... ℥ j.

Aquæ,.................. ad ʒ ij.

M. Sig:—A tablespoonful at intervals of one to four hours until relieved.—GERMAIN SEE, *Annual Univ. Med. Sci.*

### ASTHENIA.

℞. Quiniæ sulphat.........gr. xxx.
Acidi sulphur. dil.......q. s.
Aquæ.................. ℥ ij.
Tinct. ferri mur......... ℥ ss.
Spts. chloroformi....... ʒ vj.
Glycerinæ. .........ad ℥ iv.
M. Sig:—A teaspoonful three times daily.—LOOMIS.

